# Side-locked headaches: an algorithm-based approach

**DOI:** 10.1186/s10194-016-0687-9

**Published:** 2016-10-21

**Authors:** Sanjay Prakash, Chaturbhuj Rathore

**Affiliations:** 1 Department of Neurology, Smt. B. K. Shah Medical institute and research Centre, Sumandeep Vidyapeeth, Piparia, Waghodia, Vadodara, 391760 Gujarat India; 2 Department of Neurology, Smt B. K. Shah Medical institute and research Centre, Piperia, Waghodia, Vadodara, 391760 Gujarat India

**Keywords:** Side-locked headache, Unilateral headache, Trigeminal autonomic cephalagias, Hemicrania continua, Cluster headache, Paroxysmal hemicrania, Neuralgias

## Abstract

The differential diagnosis of strictly unilateral hemicranial pain includes a large number of primary and secondary headaches and cranial neuropathies. It may arise from both intracranial and extracranial structures such as cranium, neck, vessels, eyes, ears, nose, sinuses, teeth, mouth, and the other facial or cervical structure. Available data suggest that about two-third patients with side-locked headache visiting neurology or headache clinics have primary headaches. Other one-third will have either secondary headaches or neuralgias. Many of these hemicranial pain syndromes have overlapping presentations. Primary headache disorders may spread to involve the face and / or neck. Even various intracranial and extracranial pathologies may have similar overlapping presentations. Patients may present to a variety of clinicians, including headache experts, dentists, otolaryngologists, ophthalmologist, psychiatrists, and physiotherapists. Unfortunately, there is not uniform approach for such patients and diagnostic ambiguity is frequently encountered in clinical practice.

Herein, we review the differential diagnoses of side-locked headaches and provide an algorithm based approach for patients presenting with side-locked headaches. Side-locked headache is itself a red flag. So, the first priority should be to rule out secondary headaches. A comprehensive history and thorough examinations will help one to formulate an algorithm to rule out or confirm secondary side-locked headaches. The diagnoses of most secondary side-locked headaches are largely investigations dependent. Therefore, each suspected secondary headache should be subjected for appropriate investigations or referral. The diagnostic approach of primary side-locked headache starts once one rule out all the possible secondary headaches. We have discussed an algorithmic approach for both secondary and primary side-locked headaches.

## Introduction

The location of pain is an important point to be considered while making a diagnosis of headache [1]. The side of pain can be described as always on one side (side-locked headache), side shifting unilateral, unilateral alternating with bilateral, always bilateral and bilateral with more intense pain on one side [2]. Making the correct diagnosis of side-locked headaches is very important for various reasons. A few side-locked headaches may have serious underlying pathologies and early diagnosis is paramount to prevent severe complications [2]. On the other hand, some side-locked headaches respond only to a specific drug [3]. Therefore, a correct diagnosis is essential. However, the differential diagnosis of side-locked headaches is very demanding and a diagnostic confusion is frequently encountered in clinical practice.

A good knowledge and a sound diagnostic approach are essential things for the optimal management of side-locked headaches and facial pain. However, the literature is sparse regarding the diagnostic distribution and approach for side-locked headaches. The differential diagnosis of side-locked headache is wide and very long. Therefore, an orderly approach will be more appropriate for patients having pain always on the same side.

## Review

This is a narrative, clinically orientated review on the side-locked headaches and facial pain. The review is intended for all health-care specialists who treat headache patients. The diagnostic algorithms described here have not been validated but represents authors’ personal experience and views for the side-locked headaches. This review / algorithm will focus on (i) how to diagnose primary headaches and neuralgias presenting as side locked headaches (ii) when and how to investigate these patients to rule out or confirm secondary headaches (iii) when to refer these patients to other specialities.

**Fig. 1:**
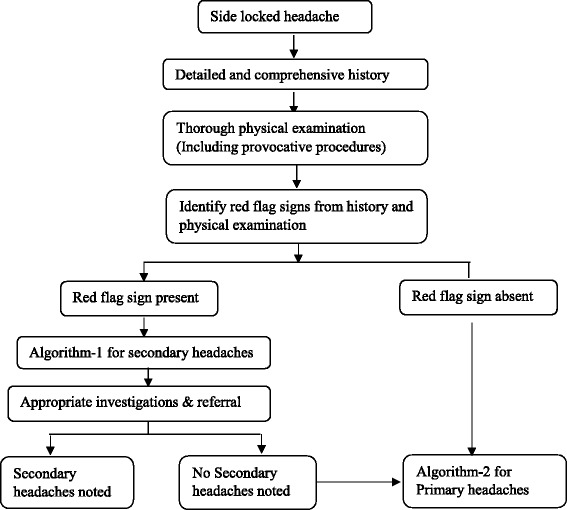
Overview of the diagnostic approach for patients with side locked headaches

**Fig. 2:**
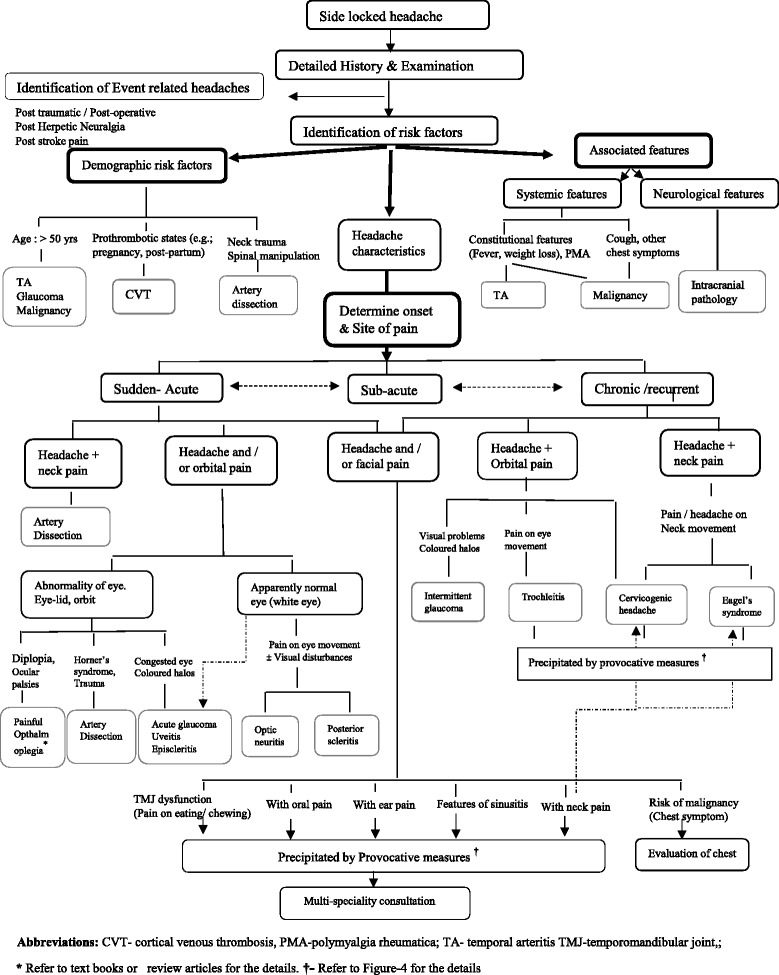
(Algorithm-1): An approach to look for the secondary causes in patients with Side Locked Headaches

**Fig. 3:**
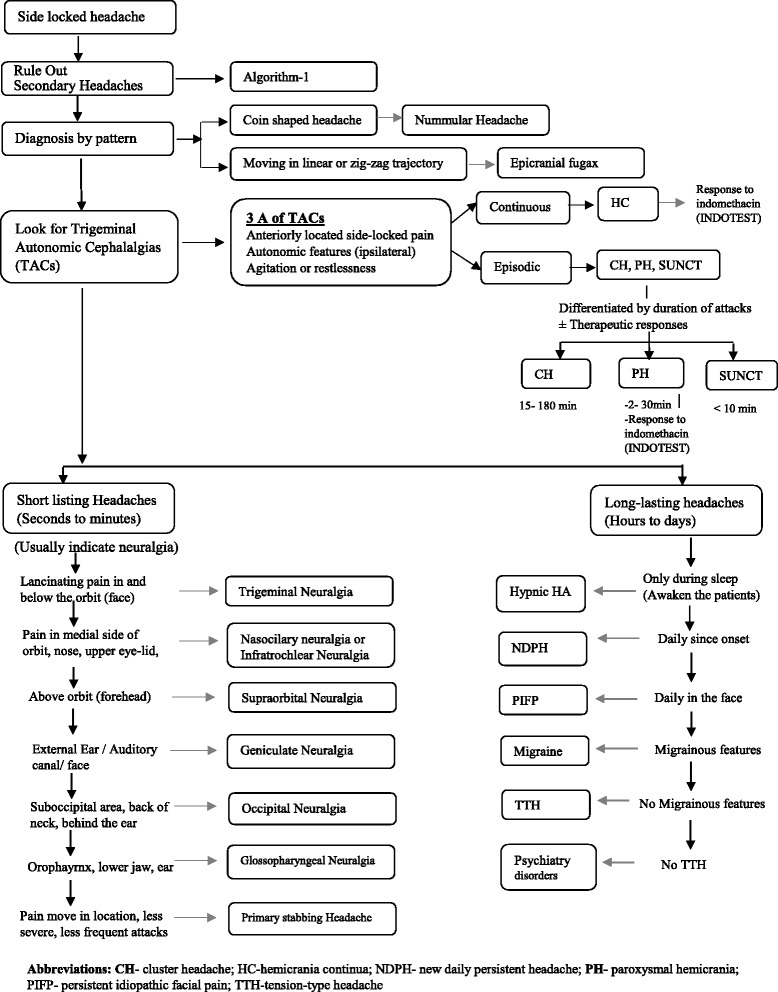
(Algorithm 2): An approach for Side Locked Primary Headaches and Neuralgias

The Fig. 1 gives the overview of the approach for such patients. The Fig. 2 (algorithm-1) highlights the approach for secondary side-locked headaches. The Fig. 3 (algorithm-2) highlights the approach and diagnosis of various primary headaches and neuralgias. Headaches related to psychiatry disorders are secondary headaches where the diagnosis is made once other secondary and primary headaches are ruled out. That is why it has been discussed in algorithm −2 with primary headaches.

## Definition

Side-locked headache literally means headache always on the one side. However, Leone et al. have suggested to quantify the frequency of unilateral headaches to label it as side-locked. They suggested that patients having more than 90 % headaches on the one side can be called as ‘side-locked’ headaches [4].

Facial pain syndrome and headache disorders are usually discussed together as both share a large number of common aetiologies [5]. A few headache disorders may have pain in the oral cavity and neck. On the other hand, a few pathologies of the oral cavity and neck may produce pain in the head and face [2]. Herein, we will include all such cases that have such overlaps.

## Epidemiology

There is a paucity of studies on the epidemiology of side-locked headache. Sjaastad and Bakketeig looked for the rare unilateral headaches (excluding common headaches such as migraine, cluster headache, and cerviocogenic headaches) in the general population in the Vågå study. They noted 53 patients (2.9 %) with rare strictly unilateral headache in 1838 parishioners [6]. There are two clinic-based studies on the strictly unilateral headaches. In a study by Ramon et. al conducted in a headache clinic, the prevalence of side-locked headaches was 18.9 % of all headaches. [7]. We found a prevalence of 19.2 % of all headaches in the Neurology clinic [2]. Thus, approximately about 20 % of patients presenting with headaches have strictly unilateral headaches.

## Etiology

- **Primary headaches**
  The prevalence of side-locked headache in the various primary headaches is summarized in Table 1. Strictly unilateral head pain is an essential feature of all trigeminal autonomic cephalalgias (TACs). The International classification of headache disorders (ICHD-3β) criteria of all five TACs incorporate “unilateral pain” as one of the must feature [3, 5]. About 85–90 % of TACs are “side-locked” [8]. Besides TACs, nummular headache is another primary headache which is by definition unilateral and side-locked [5]. Epicranial fugax is a recently described primary headache. It is also always unilateral [5]. About 56 % patients with primary stabbing headache and upto 45 % patients with hypnic headache may have side-locked headaches [9, 10]. Various studies suggest that 40–80 % migraineurs may have unilateral headache. However, a very few studies have assessed the side-locked character in migraineurs. Sjaastad et al. [11] observed 31 patients with classic migraine. They noted strict unilaterality in 42 % and unilaterality combined with bilaterality in other 42 % patients. Twenty-two patients were reassessed after 3–9 years of follow up. Strict unilaterality was constant. However, most patients showed side-shifting unilateral headaches. Only two patients showed side-locked headaches. However, Da Silva, et al. [12], after review of the literature suggested that 26 % migraineurs may have side-locked headache. Tension-type headache (TTH) and new daily persistent headache (NDPH) are largely bilateral headaches and are generally not considered in the differential diagnosis of side-locked headaches. However, the literature review suggests that a subset of patients with NDPH (11–18 %) and TTH (4–36 %) may have side-locked headaches [2, 12]. Even rare primary headaches may have isolated case as side-locked headaches [2].

**Table 1:** Proportion of patients with Side-locked pain in different primary headache disorders

Primary headaches	(%) of side locked headaches
Migraine	17–31 % [1]
Tension-type headache	4–36 % [1]
Cluster headache	69–92 % [3]
Paroxysmal Hemicrania	85–97 % [40]
SUNCT/SUNA	80–88 % [1, 3]
Hemicrania continua	92–100 % [41]
Primary stabbing	22–56 % [9]
Nummular headache	93–100 % [5, 45]
Hypnic headache	22–45 % [49]
NDPH	11–18 % [2]
Epicranial Fugax	~100 % [10]
- **Secondary headaches**
  The common secondary causes of side-locked headaches are summarized in Table 2. It has been arranged according to the ICHD-3β classification system [5]. Group-6 and group-11 are very important for side-locked headaches. Group-6 is about “the headache attributed to cranial or cervical vascular disorder”. Cervical artery dissection, temporal arteritis (TA) and cerebral venous thrombosis (CVT) are few main pathologies in this group. Approximately two-thirds of patients with cervical artery dissection may have a unilateral headache. A headache may be the initial symptoms in about half of the patients with cervical artery dissection [13]. Headache is the most common initial symptoms in CVT. It is usually diffuse. However, lateral sinus thrombosis may present with a headache on one side only [14]. TA may produce diffuse and bilateral headache. However, unilateral headache is the most common initial symptom [15].

**Table 2:** Causes of secondary side locked headaches and neuralgias (according to the ICHD-3β) [5]

ICHD-3β code	Diseases
The secondary headaches	
5. Headache attributed to trauma or injury to the head and/or neck	Post traumatic, Post craniotomy,
6. Headache attributed to cranial or cervical vascular disorder	Temporal Arteritis, carotid or vertebral artery dissection, cerebral venous thrombosis, arteriovenous malformation, unruptured aneurysm, Post-endarterectomy headache, intracranial endovascular procedure
7. Headache attributed to non-vascular intracranial disorder	intracranial neoplasia (especially pituitary and CP angle tumor, Chiari malformation 1,
10. Headache attributed to disorder of homoeostasis	Airplane travel headache, Cardiac cephalalgia
11. Headache or facial pain attributed to disorder of the cranium, neck, eyes, ears, nose, sinuses, teeth, mouth or other facial or cervical structure	Cervicogenic headache, glaucoma, ocular inflammatory disorder, trochleitis, disorder of the ears, rhinosinusitis, teeth or Jaw, temporomandibular disorder, inflammation of the stylohyoid ligament.
12. Headache attributed to psychiatric disorder	Somatization,
Painful cranial neuropathies, other facial pains and other headaches	
13. Painful cranial neuropathies and other facial pains	Trigeminal neuralgia, Painful trigeminal neuropathy (e.g. Acute Herpes Zoster, Post-herpetic), Glossopharyngeal neuralgia, nervus intermedius neuralgia, Occipital neuralgia, Optic neuritis, ischaemic ocular motor, nerve palsy, Tolosa-Hunt syndrome, Paratrigeminal oculosympathetic (Raeder’s) syndrome, Recurrent painful ophthalmoplegic neuropathy, Persistent idiopathic facial pain, Central post-stroke pain.
  Group −11 of ICHD-3β classification includes “Headache or facial pain attributed to disorder of the cranium, neck, eyes, ears, nose, sinuses, teeth, mouth or the other facial or cervical structure”. One of the criteria of most of the headache disorders in this group is either unilateral headache or pain localized to the site of the lesion [5]. One important subgroup in this is the headache related to ocular and orbital causes, and it may present as painful ophthalmoplegia. A few cranial neuralgias/neuropathies may also present as painful ophthalmoplegia. Painful ophthalmoplegia may have serious underlying pathologies. However, the list of the causes of painful ophthalmoplegia is very long. Clinicians are encouraged to look elsewhere for the details of painful ophthalmoplegia [16].
  - **Painful cranial neuropathies/neuralgias and other facial pains**: This group (group-12 of ICHD-3β) largely includes strictly unilateral painful syndrome. Neuralgias are by definition strictly unilateral. Neuralgias may be both, primary and secondary. Persistent idiopathic facial pain (PIFP), recurrent painful ophthalmoplegic neuropathy, Tolosa-Hunt syndrome, ischaemic ocular motor nerve palsy, etc. are described here [5].

### Diagnostic distribution of different side-locked headaches in the clinic

There are only two clinic-based studies on side-locked headaches. Table 3 shows the distribution of common causes of side-locked headaches after the pooled analyses of both studies (*n*-407 cases). There were a total 41 different diagnoses in these 407 patients. Primary headaches constitute 61 % of all side locked headaches. About one-third patients had either secondary headaches or neuralgias. Cluster headache (CH) is the most common side-locked headache (20 %) in the clinic setting. Overall TACs constitutes one-third of total side-locked headaches. Migraine (14 %) was the second most common side-locked headache. Other common primary headache disorders were hemicrania continua (7.3 %), tension-type headache (4.2 %), SUNCT/SUNA (4.2 %), nummular headache (3.4 %), paroxysmal Hemicrania (3.2 %), NDPH (2.2 %), and primary stabbing headache (1.2 %).

**Table 3:** Diagnostic distribution of different types of side locked headache in the clinic setting (after the pooled analyses of two studies) (*n*-407 cases) [2, 7]

Disease	Patients (%)
Primary headaches	61.7
Trigeminal Autonomic Cephalalgias	34.7
Cluster headache	19.9
Migraine	14.0
Hemicrania continua	7.3
Tension-type headache	4.2
SUNCT/SUNA	4.2
Nummular headache	3.4
Paroxysmal Hemicrania	3.2
New Daily Persistent Headache	2.2
Primary stabbing	1.2
Secondary headaches & Neuralgias	34 % (20/14)
Cervicogenic headaches	8.1
Related to psychiatry disorders	4.2
Trigeminal Neuralgia	3.9
Persistent idiopathic facial pain	2.5
Post herpetic neuropathy	2.2
Temporomandibular joint (TMJ) disorders	1.7

(Headache disorders with a prevalence of more than 1 % have been included in the table)

Cervicogenic headache (CEH) is the most common secondary headache presenting as side-locked headache (8.1 %). A headache related to psychiatry disorders (4.2 %) and temporo-mandibular joint (TMJ) dysfunctions (1.7 %) are two other common secondary headaches. Trigeminal neuralgia (3.9 %) is the most common neuralgia. Persistent idiopathic facial pain (2.5 %) and post-herpetic neuralgia (2.2 %) are other common causes of side-locked facial pain.

## Diagnostic approach for side-locked headaches

### Overview of the approach

Figure 1 provides a stepwise diagnostic scheme for the side-locked headaches.

- Step 1: An accurate and comprehensive history.
- Step 2: A thorough physical examination (including provocative measures).
- Step 3: Interpretation of the findings. Identify red flag signs from the history and examinations (Table 4)

**Table 4:** Red flag signs pertinent with side-locked headaches

Mnemonic (SNOOP4) [17]	Clinical descriptions	Secondary headaches
Systemic	Fever	Temporal Arteritis (TA), malignancy, infective pathology (sinus, eye, teeth, etc.)
	Weight loss	TA, malignancy,
	Cough and other chest symptoms	Carcinoma lung
	Nasal symptoms	Sinus related headaches
Neurological	Opthalmoplegia (diplopia, ocular palsies)	Painful opthalmoplegia syndrome
	Visual disturbances	Ocular (glaucoma, post sclertitis, other inflammatory pathologies), Optic Nerve (optic neuritis, TA), orbital causes.
	cognitive, motor, sensory or cerebellar abnormality	Intracranial pathologies
Onset sudden	Peak within minutes	Cervical artery dissection
Onset after 50 years	New headache in elderly	TA, Malignancy, Glaucoma (after 40 years, acute or intermittent), cervicogenic headache
Pattern of headaches	Persistent & progressive	A large number of secondary headaches have persistent & progressive course
	Pain other than head (i.e. headache with pain in eye, face, and neck)	Look for the pathologies at the site of maximum pain
	Precipitated by provocative maneuvers	Referred to Fig. 4
	papilledema	Intracranial pathologies
- Step 4: Formulate diagnosis according to the red flag signs. It will help to select investigations or referral to confirm or rule out secondary headaches (Fig. 2, Algorithm-1)
- Step 5: If no red flag signs or no secondary headaches detected, look for primary headaches or neuralgias (Fig. 3, algorithm-2)

**Fig. 4:**
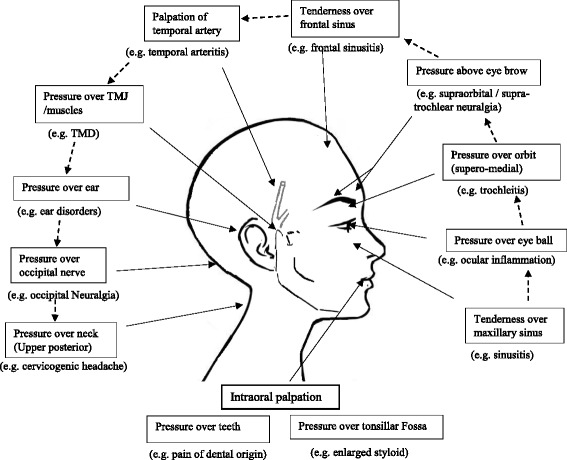
Provocative procedures to reproduce pain in various secondary headaches and Neuralgia
- **Step-3: Identification of red flag signs on the basis of history and physical examinations**
  Classically red flag signs are described with the mnemonics of SNOOP4 [17]. It is also useful in patients with side locked headaches. Table 4 summarize the red flag signs pertinent with side-locked headaches.
- **Step-4: Formulating a possibility of secondary headaches on the basis of red flag sings**
  Figure 2 (algorithm-1) provides a stepwise approach to look for the secondary causes of side-locked headaches. This algorithm for secondary headaches just guides the clinicians to select investigations or referral to confirm or rule out secondary headaches.
  The most important point in patients with side-locked headaches is to find out serious underlying pathologies. Vascular pathology (TA, CVT, and Dissection) ocular and optic pathology (painful opthalmoplegia, glaucoma, optic neuritis, etc.), intracranial pathologies and malignancies are a few serious underlying pathologies. Early detection is essential to prevent serious complications. Most of the secondary headache have some red flag signs. However, the patients may have just side-locked headache in the early stage. Therefore, a high index of suspicion is essential at every step. Following steps can be used for excluding secondary headaches
  - **(I). Start having suspicion with the demographic profiles of the patients**
    Age (>50 years) is the strongest risk factor for the suspicion of TA, Glaucoma, and malignancy. The patients should be enquired about the associated clinical features pertinent to these disorders and should be investigated accordingly. Prothrombotic state is a red flag signs for all new headaches, even side locked headaches. Prothrombotic states are a risk factor for CVT. A recent history of neck trauma or spinal manipulation may be an indication for cervical artery dissection [18].
  - **(II). Associated features (constitutional and Neurological symptoms)**
    The presence of constitutional symptoms (fever, weight loss, and polymyalgia) again suggest a possibility of TA and malignancy. Neurological features may suggest intracranial pathology.
    - *Temporal Arteritis (TA)*: TA should be suspected in every elderly patient (>50 years). The presence of constitutional symptoms increases its possibility. A history of jaw claudication, visual disturbances, and the scalp or temporal artery tenderness are a few specific features for TA. Elevated erythrocyte sedimentation rate (ESR) (>50 /h) and C-reactive protein (CRP) (>50 mg/L) increases the probably of TA. Temporal artery biopsy is the gold standard test and should be considered on the suspicion of TA. Non-invasive investigations (duplex sonography, high-resolution magnetic resonance imaging, positron-emission tomography) can also be undertaken [19].
    - *Glaucoma*: it usually starts after 40 years of age. The patient may have unilateral headaches (with eye pain), photophobia, nausea, and vomiting. It may mimic an attack of migraine. The presence of redness of eye, tearing, halos around light should warn the patients and clinician about the possibility of acute angle-closure glaucoma (ACG). Acute ACG is an ophthalmic emergency and patients should be immediately referred to ophthalmologist for further managements [20].
      Sub-acute angle-closure glaucoma may also cause migraine like headache. Shindler et al. [21] reported 11 elderly patients (mean age-54 years) with a long history (average 2.6 years) of recurrent unilateral headaches (at least 2 attacks/ week). All patients fulfilled the criteria of migraine except for the duration of the headache attacks (none had headache longer than 4 h). The patients didn’t have much benefits from anti-migraine therapies. The patients were later diagnosed as having intermittent angle closure glaucoma. Ten patients underwent for laser peripheral iridotomy (LPI). All 10 patients had improvement of their headaches (6 patients -complete resolution and 4 patients-significant improvement). The only features atypical for migraine was headache attack duration (never more than 4 h). Atypical migraine or a change in the pattern of migraine headaches or new onset migraine after the age of 40 years should always be evaluated for the presence of acute or sub-acute ACG.
      Acute ACG may be precipitated by certain drugs, including drugs used for migraine (e.g. topiramate). Therefore, any change in the headache pattern after starting topiramate and other drugs should warn clinicians and patients about the possible development of acute glaucoma [22].
    - *Malignancy*: Intracranial tumour may cause side-locked headache. However, the diagnosis of this is not difficult as most of them will cause some neurological symptoms and can be detected on neuroimaging. The problem arises with the malignancy which causes referred pain in the head and face [23]. The hemicranial pain may be the first symptom of carcinoma lung. It is a rare condition. But, the diagnosis should not be delayed, because of the morbidity and mortality associated with the condition. The condition may mimic TA (weight loss, fever, elevated ESR), hemicrania continua (HC), persistent idiopathic facial pain (PIFP) and many other primary and secondary headaches.
      Eross et al. [23] reviewed the 30 such cases and suggested a triad for the suspicion of hemicranial pain because of lung mass: a history of smoking (100 %), periauricular pain (84 %) and an elevated ESR (77 %). Other features that may suggest malignancy are constitutional features (fever, weight loss, cough) (70 %) and clubbing (33 %). All such patients should be screened for carcinoma lung.
  - **(III). Categorize patients according to headache characteristics**
    The approach for other side-locked headaches largely depends on the headache onset (or pattern of the headache) and the site of maximum pain. Headache onset (or pattern) can be classified as acute (<1 month), sub-acute (1–3 months) or chronic (>3 months). Most serious underline causes have sudden or acute onset pain. Pain can be further classified on the basis of the site of pain or site of maximum pain. Headache can be associated with orbital pain, orofacial pain and neck pain. The involvement of the particular site may give hint for an underlying pathology.
    - **(a) Acute hemicranial pain**
      Sudden or acute headache is probably the most important red flag sign for side-locked headache. A possibility of cervical artery dissection should always be considered in all acute or sudden hemicranial headaches, because of the risk of morbidity and mortality associated with delayed diagnosis of this condition. It may cause pain at any site including neck, occiput, frontotemporal region, orbit, face, teeth or ear. The patients should be enquired about the other risk factors and clinical characteristics of dissection (Horner’s syndrome, cerebral or retinal ischemia and a history of neck trauma or cervical manipulation) [13].
      Bedsides artery dissection, there are many other serious causes for acute hemicranial pain. The diagnosis of such conditions can be facilitated by considering the site of maximum pain.
    - **(b) Acute orbital pain with headache or without headache**
      It should be evaluated very carefully as there are many underlying serious pathologies. The patients with orbital pain should be examined carefully for ocular palsy, eyelid abnormality (swelling, drooping), conjunctival injection (red eye), visual disturbances, and pain on eye movement [24, 25].
      - **(i) Orbital pain in association with ocular motor nerve palsies**
        It is the clinical hallmark of the painful ophthalmoplegia syndrome. The patients may have associated eye-lid oedema and proptosis. Painful opthalmoplegia is a medical emergency [16] and immediate imaging is mandatory. For further details, the readers are encouraged to check relevant monographs on painful opthalmoplegia.
      - **(ii) Redness of eye ball (conjunctival injection)**
        Red eye may be the part of painful opthalmoplegia. However, it may be the indicative of glaucoma and local ocular inflammation (irritis, uveitis, conjunctivitis or episcleritis). Urgent ophthalmic referral is recommended if patients have associated visual abnormalities (including vision loss and coloured halos) to look for a possibility of glaucoma, episcleritis or uveitis [25].
      - **(iii) Orbital pain with Horner’s syndrome**
        In this case one should suspect the presence of cervical artery dissection [13].
      - **(iv) Orbital pain with normal eye (white eye)**
        All above mentioned causes may have normal opthalmoplegic examinations. Therefore, a close watch is essential for the all patients having new onset persistent orbital pain [24]. In doubtful cases, urgent neuroimaging of orbit and ophthalmic referral is advisable. However, a few diseases with orbital pain such as optic neuritis and posterior scleritis may really have white eye (no apparent abnormality). The patients may have subtle vision loss which they may not be aware of. Moreover, optic neuritis (and even posterior scleritis) may have isolated side-locked headache as initial manifestation [26]. Ophthalmic examinations (including fundoscopy) may be normal. The diagnosis at the juncture may have paramount effects on the prognosis. Pain on eye movement may be the only clue on early stages. Contrast MRI of optic nerve and eye ball may pick up optic neuritis and posterior scleritis respectively [25, 26].
    - **(c) Acute headache with neck pain**
      Acute / recurrent headache with predominantly neck pain is more common with primary headaches (especially migraine and TTH). However, many nonspecific causes, such as, strain, or spasm of the neck muscles may cause neck pain with headache. The most important diagnosis to be considered here is cervical artery dissection [13].
    - **(d) Chronic headache with orbital pain**
      Contrary to acute causes, there are very limited secondary causes of chronic headache with orbital pain and majority of these patients have primary headaches. In this scenario, age more than 40 years suggest a possibility of secondary headaches. The following conditions should be considered in patients with chronic headache with orbital pain.
      - *Sub-acute angle-closure glaucoma*: As discussed above, sub-acute ACG may closely mimic migraine. Intermittent visual disturbances with sub-acute ACG may mimic migrainous visual aura. It may remain undiagnosed for years. Side locked chronic migraine (may be even daily for years) in elderly should be evaluated on this line. Headache duration of less than 4 h and poor response to anti-migraine therapies should warn clinicians for the presence of subacute ACG [21].
      - *Cerviocogenic eye pain*: Cervicogenic headache (CEH) predominantly causes neck pain that radiate to involve the whole head and even orbit. However, in a subset of patients with CEH, the pain may be localized to the orbit only [25]. Elderly patients with chronic orbital pain should also be examined for the presence of cerviocogenic headaches.
      - *Trochlear headache / trochelitis*: it may cause chronic hemicranial headache with maximum pain in the orbit. It will get aggravated by eye movement (especially supraduction). There will be tenderness or aggravation of pain by pressing on the upper-inner angle of the orbit [27].
    - **(e) Chronic Headache with neck pain**
      Age more than 40 years and neck pain getting aggravated by neck movements are red flag signs for secondary headaches. Cervicogenic headache and elongated styloid process (Eagle’s syndrome) are two important causes in this group. *CEH*: It was the most common secondary hemicranial headaches in both cross sectional studies on the side-locked headaches [2, 7]. Various observations have shown that pain from upper cervical joints and muscles can be referred to the head, orbit and face. This pain is similar to the pain felt in the shoulders, upper limbs, trunk, or lower limbs that is referred from spinal sources [28]. There is convergence between cervical and trigeminal afferents in the trigeminocervical nucleus. It allows the bidirectional referral of pain between the neck and the head and face. The C2–3 zygapophysial joints are the most common site of CEH [28]. Recent observations suggests that upper cervical radiculopathy may be an important cause of CEH [5].
      Laterality is not mentioned in the ICHD-3β diagnostic criteria for CEH. However, Cervicogenic Headache International Study Group (CHISG) criteria for CEH suggest that unilateral head pain, without side shift is a fundamental quality of CEH [29]. Therefore, a suspicion of CEH should be done in all side-locked headache. A possibility of CEH is increased when pain starts in the neck and radiates anteriorly. Aggravation of symptoms by abnormal postures or neck movement further increases the possibility of CEH. Headache is usually precipitated by pressure or palpation. There may be history of whiplash injury [28]. A temporary relief following diagnostic blockade of a cervical structure or its nerve supply may be a clue for the presence of CEH [5, 29].
      - *Elongated styloid process (Eagle’s syndrome)*: this is a rare syndrome. However, the patients may have widespread hemicranial (even bilateral) pain. The pain may be present in the head, neck, face, periauricular, ear, and even in the oral cavity. The patients may have a unique combination of pain getting aggravating by (i) neck movement and (ii) swallowing or chewing. The patient may also have dysphagia, odynophagia, hypersalivation, and foreign body sensation in the pharynx. There may be a history of tonsillectomy. Digital palpation of the styloid process in the tonsillar fossa will precipitate pain and it is an important clinical clue. In such cases, X-ray or CT scan targeting the styloid process should be undertaken. [30].
    - **(f) Headache with facial pain (Acute and chronic)**
      Orofacial pain with or without a headache is a complex symptom that includes a myriad of etiologic possibilities. It may arise from intracranial structures, cranial bones, neck, eyes, ears, temporomandibular joint, sinuses, facial tissues, oral structures or cervical arteries [31].
      Sudden severe pain in the face (with or without headache and neck pain) points a possibility of cervical artery dissection and it should be dealt with carefully. However, all other structural lesions or causes may lead to both acute and chronic painful condition. The diagnosis is mainly facilitated by the site of maximum pain, aggravating factors, associated symptoms and precipitation by provocative measures [31, 32].
      - *Temporomandibular joint (TMJ) dysfunction*: In this condition, pain is maximum in the area anterior to ear. The pain usually gets aggravated by chewing and eating. There will be clicking, popping, or crepitus on the movement of temporomandibular joint. Jaw movement is usually limited. The headache is exacerbated by jaw movements, or by pressure on the TMJ and surrounding muscles of mastication [33, 34].
      - *Sinusitis*: It usually causes acute pain. However, chronic pan because of chronic sinusitis is also a possibility. Patients may have history suggestive of rhinosinusitis (fever, nasal congestion, nasal discharges). Pressure over particular sinus usually precipitates the pain [5].
      - *Dental origin*: Pain of dental origin can present as both I both acute and chronic pain. It is suspected when the patients have maximum pain in the oral cavity. Pressure on the culprit teeth may precipitate the pain [5, 32].
      - *Pain related to ear*: Inflammatory and other conditions of ear may cause acute and chronic pain. Pressure over ear may induce pain in these conditions [5].
      - *Facial pain related to neck pathology*: Neck should always be examined in patients presenting with facial pain. [28].
      - *Facial Pain related to malignancy*: it is discussed above. Facial pain may the first symptom of carcinoma lung. Acute facial pain in the elderly smoker raise a possibility of this dangerous entity [23].
- **Step-5: Approach for primary headaches**
  - **(I). Rule out secondary side locked headaches**
    Side-locked headaches is itself a red flag sign [6]. Even if there is no other red flag sign, MRI brain should be done in all side-locked headaches. Secondary TACs almost mimic primary TACs [35, 36]. Side-locked migraine is not a typical migraine. Side locked migraine with auras may mimic occipital AVM [37]. Classical TN is usually caused by a vascular loop abutting the trigeminal nerve. Hence, the diagnostic approach of primary side-locked headache starts once one has ruled out all the possible secondary headaches, including intracranial lesions.
  - **(II). The diagnosis by the pattern**
    Nummular headache and epicranial fugax (EF) are recognized by their pattern. An awareness of this syndrome is enough to clinch the diagnoses.
    - *Nummular headache*: it is characterized by focal and well-circumscribed pain fixed within a rounded or elliptical-shaped area of the scalp. Patients often delineate the outline of the headache area with a finger. The headaches are typically unilateral, side-locked, and fixed in the location [5].
    - *Epicranial Fugax*: it is characterized by brief paroxysms of pain that starts at one point and moves in a linear or zigzag territories in one hemicranium. Starting and terminating points belong to two different nerves territories. Attack duration varies between 1 and 15 s. Attack frequency is extremely variable and ranges from several attacks per day to just a few attacks in a year [10].
  - **(III). Recognizing TACs**
    TACs are a group of 5 different primary headaches: cluster headache (CH), paroxysmal hemicranias (PH), short-lasting unilateral neuralgiform headache with conjunctival injection and tearing (SUNCT), Short lasting unilateral neuralgiform headache attacks with cranial autonomic symptoms (SUNA), and hemicrania continua (HC) [5]. About one-third of the side-locked headaches belongs to TACs [2]. TACs are known for their severity and response to a specific drug. Therefore, it should be considered first in any diagnostic approach for side-locked headaches.
    All TACs have well-defined clinical picture. However, most TACs remain undiagnosed for years [38, 39]. Even a typical case of TACs is easily missed out by physicians. Unawareness to TACs is the main reason for delayed diagnosis. Various authors have recommended continuous medical education or training for the general neurologists and other clinicians who care headache patients. TACs are frequently misdiagnosed as migraine, trigeminal neuralgia and sinusitis [38, 39]. Therefore, in any side-locked headaches, TACs should be considered with a priority.
    All five TACs share three common features. Unilateral pain in the trigeminal distribution is the central features of all TACs. Two other associated features of all TACs are cranial autonomic features and agitation during attacks/exacerbations. These three symptoms can be memorized easily by a mnemonic- **3A**. **3A** includes: (i). Anteriorly located (orbital, frontal) unilateral pain (ii) Autonomic features in the same area (ipsilateral) during attacks / exacerbations. (iii) Agitation during attacks or exacerbations. Although it is not validated, the presence of all 3A is highly suggestive of TACs (especially when one has ruled out secondary headaches). With this mnemonic, the clinicians may easily familiarize themselves with TACs.
    TACs should be suspected in all patients who have anteriorly located pain. A few percentages of patients may not have either agitation or autonomic features. The autonomic features are universal in SUNCT/SUNA. It is noted in more than 90 % cases of CH and PH [3, 40]. The prevalence of cranial autonomic features in HC varies between 63–97 % [41]. In the same way, more than 90 % with CH and PH may have agitation during the attacks. However, only two-third patients of HC and SUNCT/SUNA will have agitation [3, 41]. Therefore, even 2A is highly suggestive of one of the TACs. In fact, the presence of either agitation or autonomic features alone is enough to fulfil the ICHD-3β criteria of CH and HC [5]. However, one should be careful while interpreting cranial autonomic features (CAS), as it may present in migraine and other headaches. Various studies indicate that about 56 % migraineures may have at least one autonomic features [42]. However, CAS in migraine are usually bilateral, mild to moderate in intensity and not very consistent with headache attacks [42].
    Once you recognize the presence of 3A, the individual TAC is recognized on the basis of the pattern of headache, frequency and duration of the attacks [5]. On the some occasions, a therapeutic trial is also essential to differentiate to various TACs. All TACs are episodic except HC. HC will have continuous background pain with variable episodic exacerbations. Therefore, if the patient has 3A, first rule out HC, and ask about the continuous background pain. Response to indomethacin will confirm HC. Indomethacin is usually started at the dose of 25 mg three times in day. Most patients show marked improvement within 48 h of initiation of oral indomethacin [41]. Although a few patients may show delayed response. In atypical cases, injectable indomethacin (50 mg IM) (INDOTEST) can be given to predict the response of oral indomethacin [43]. All other TACs are episodic, which differ in attack duration and frequency. CH has the longest attack duration (15–180 min) and relatively low attack frequency. PH has intermediate duration (2–30 min) and intermediate attack frequency. SUNCT and SUNA have the shortest attack (1–600 s) duration and the highest attack frequency [5]. PH shows overlap in duration with both CH and SUNCT/SUNA. However, PH shows dramatic response to indomethacin. IDOTEST can be done to differentiate borderline cases of PH [44]. The patients with PH will show protective effects of injectable indomethacin. SUNCT/SUNA also needs to be differentiated with trigeminal neuralgia (discussed below).
  - **(IV). Categorize headache as short lasting (less than a few minutes) and long lasting**
    - **Short-lasting headaches attacks**
      Short lasting headaches of less than a few minutes are highly suggestive of neuralgias. The diagnosis of neuralgias are made according to character of pain (lancinating pain for a few seconds to a few minutes), site of pain, and aggravation by certain manoeuvres [45]. Figs. 3 and 4 may help you to differentiate various types of neuralgias.
      - *Trigeminal Neuralgia*: TN is the most common neuralgia. TN usually involves maxillary division of trigeminal nerve. A few percentages of patients may have involvement of ophthalmic division. Ophthalmic TN should be differentiated with SUNCT/SUNA. The presence of refractory period, no or very mild autonomic features and response to carbamazepine favour a diagnosis of TN [46].
      - *Occipital neuralgia*: A diagnosis of occipital neuralgia is very important as it may mimic many primary and secondary headaches. There may be constant pain between the paroxysms. The pain is felt even in the retro-orbital area. The pain is usually exacerbated by the flexion of the neck. The associated symptoms with neuralgia includes visual disturbances (67 %), tinnitus (33 %), dizziness (50 %), nausea (42 %), and even autonomic features like the congested nose (17 %). There may be even photophobia and phonophobia [47]. Therefore, the symptoms complex of occipital neuralgia overlap with migraine, TTH, cluster headache, hemicrania continua, and cervicogenic headaches. The greater occipital nerve (GON) is more frequently involved (90 %) as compared with the lesser occipital nerve (LON) (10 %). Triggering of attacks by pressure over the nerves may the best clue for occipital neuralgia [47, 48]. The greater occipital nerve is usually palpated 2–3 cm lateral and inferior to the occipital protuberance or in the depression just lateral to the insertion of the trapezius muscle into the occipital bone [47]. A temporary improvement by anaesthetic block of the nerve may confirm the diagnosis [5].
      - *Primary stabbing headaches*: it should be considered in all short-lasting headaches. It is an underrecognized primary headache disorder and may mimic SUNCT, TN, and other neuralgias.
        It is characterized by its ultrashort duration. The severity is much less as compared to neuralgias and SUNCT, and usually do not significantly interfere with daily life. It can occur in any dermatome and there may be series of stabs (without any refractory period). Usually, there are no precipitating factors as noted with other neuralgias. It classically responds to indomethacin [9].
**Long lasting headache attacks**
      This group of headaches has a variable pattern of headaches. Many of them classically present with diffuse and bilateral headaches. A few may have side-shifting headaches alternating with bilateral pain. Therefore, it should be diagnosed in the last.
      A headache exclusive during sleep suggests hypnic headache [49]. A daily headache since the onset suggests NDPH. Daily pain mainly in the face suggests a possibility of PIFP. After that, you should differentiate between migraine and TTH. The readers are encouraged to verify ICHD-3β diagnostic criteria before labelling it any of these primary headache disorders [5].
      If a patient does not fill any primary or secondary headache disorders, a possibility of headache related to psychiatry disorders should be considered. It was the second most common cause of secondary side-locked headaches in both clinic based studies.
      Despite this, a small proportion of patients may not be classified into any primary or secondary headache disorders and remains unclassified according to ICHD-3β diagnostic criteria.

- **Step-1: History taking**
  - (i) History targeting demographic profiles of patients
    Age, gender, risk factors for hypercoagulable states (pregnancy, post-partum state, contraceptive pill use, etc.), associated other diseases (associated malignancy, HIV infection, etc.).
  - (ii) History targeting headache characteristics
*Duration of illness*, *pattern* of headache (continuous, episodic, or continuous with episodic exacerbations), *duration* of headache attacks, *frequenc*y of episodic attacks or exacerbations, *location* of headache and *severity*.
  - (iii) History targeting associated symptoms
    (a) constitutional symptoms: fever, weight loss, polymyalgia, chest pain, cough, breathlessness, etc. (b) A history targeting intracranial pathologies - seizures, diplopia, vision loss, gait problem, sensory symptoms, etc.) (c) A history targeting migraine- migrainous features (nausea, vomiting, photophobia, phonophobia and auras) (d) A history targeting TACs- restlessness/agitation and cranial autonomic features (conjunctival injection, lacrimation, rhinorrhea, nasal congestion, ptosis, eye-lid edema, facial sweating and flushing, and sensation of fullness in the ear).
- **Step-2: Physical Examinations**
  A large number of intracranial and extra cranial abnormalities may cause side-locked headaches. Therefore, a thorough physical and neurological examinations is essential in patients with side-locked headaches. One of the characteristics features of the headache attributed to extra-cranial structures (group-11 of ICHD-3β) is that headache gets aggravated by some provocative procedures (either pressure to tissues or some other manoeuvres) [5]. A few neuralgias also gets aggravated by pressure. We suggest that examination for provocative procedures should be done on each patient with side-locked headaches as many of these headaches may closely mimic primary headaches. We suggest an orderly approach for such provocative procedures, so that it can be remembered easily and physicians can make a reflex or habit to do it in all patients with side-locked headaches. This is summarized in Fig. 4. You can start from pressure over maxillary sinus and can go in semi-circular pattern to end at testing the neck. In addition, one can palpate tonsillar fossa (for enlarged styloid -Eagles’ syndrome) and teeth.

### Limitation

There is paucity of epidemiological studies on the side locked headaches. Only two cross-sectional studies in clinics with a total 407 patients. There were a total 41 diagnosis in these two studies. So, the diagnostic distribution described here may have many limitations. Such patients are dealt with a variety of clinicians. A particular group of patients may visit specific physicians. (Patients with predominantly facial pain may visit to either otolaryngologists or dentists). Therefore, the diagnostic distribution of side-locked headaches may vary with the clinics. The diagnostic distribution may be different in the emergency setting. Although the diagnostic distribution may be different, the approach should be uniform by all clinicians.

## Conclusion

The differential diagnosis of side-locked headaches can be difficult as it includes a large number of primary and secondary headaches and cranial neuropathies. Patients may visit to a variety of clinicians including headache experts, dentists, otolaryngologists, ophthalmologist, psychiatrists, and physiotherapists. There is a need to develop a uniform approach so that patients can receive correct diagnoses in their first visit to the clinicians.
